# Shared Delusional Parasitosis in Two Families: Clinical Insights Into Folie à Deux and Folie à Trois

**DOI:** 10.2196/78398

**Published:** 2025-08-07

**Authors:** Gökçe Işıl Kurmuş, Hanife Karataş, Elif Erdem, Süheyla Doğan Bulut, Müzeyyen Gönül, Selda Pelin Kartal

**Affiliations:** 1 Department of Dermatology & Venereology, Ankara Etlik City Hospital, Varlık Street. Halil Sezai Erkut Avenue. Etlik/Yenimahalle, Ankara, 06170, Turkey, 90 3127970000; 2 Department of Psychiatry, Ankara Etlik City Hospital, Ankara, Turkey

**Keywords:** delusional parasitosis, shared psychotic disorder, folie à deux, matchbox sign, psychodermatology

## Abstract

Delusional parasitosis is a rare psychotic disorder characterized by individuals firmly believing that they are infested with parasites despite no medical evidence. It may be shared among close contacts—termed *folie à deux* when 2 individuals are affected or *folie à trois* when 3 individuals share the delusion. Delusional parasitosis’ somatic focus often leads patients to seek dermatologists, causing delayed diagnoses and unnecessary antiparasitic treatments. Herein, we present 2 familial cases of shared delusional parasitosis. In both cases, patients exhibited the matchbox sign, presenting nonparasitic materials as “evidence” of infestation. Dermatological and psychiatric evaluations excluded organic causes, diagnosing primary delusional parasitosis. Treatment with antipsychotic medications led to symptom remission. Psychoeducation was critical in preventing relapse in secondary cases. Delusional parasitosis with shared delusions is often misdiagnosed, requiring dermatologists to recognize it early. A multidisciplinary approach that combines psychiatric care and psychoeducation is essential for effective management and for preventing the reinforcement of delusional beliefs.

## Introduction

Delusional parasitosis is a rare psychotic disorder characterized by a firm and unshakable belief that one’s body is infested with parasites despite the absence of any objective medical evidence [1]. Patients with delusional parasitosis predominantly seek medical attention from dermatologists and primary care physicians rather than psychiatrists, as they firmly attribute their symptoms to a dermatological or parasitic cause [2]. The disorder is more prevalent among middle-aged and older women, with the female to male ratio being equal in individuals younger than 50 years but increasing to 3:1 in individuals older than 50 years [3].

In certain cases, delusional parasitosis is shared among close contacts, and this phenomenon is termed *shared delusional parasitosis* [1]. The transmission of delusional beliefs from one individual (primary) to a second person who has a close emotional or physical relationship with the primary individual is termed *folie à deux*. When the delusion is shared by 3 people, it is classified as *folie à trois*. Studies indicate that 5% to 15% of delusional parasitosis cases involve more than one individual (typically family members or cohabitants) [2]. In these cases, the primary affected individual—known as the “inducer”—causes another individual—known as the “recipient”—to adopt their delusional beliefs [1]. The separation of affected individuals often leads to symptom resolution in the recipient, whereas the inducer usually requires targeted psychiatric intervention [4].

The management of delusional parasitosis presents significant challenges, as most patients refuse psychiatric evaluation and resist pharmacological treatment due to their firm belief that their condition has a dermatological origin [5]. A multidisciplinary approach that incorporates dermatologists, psychiatrists, and primary care physicians is crucial for effective management [6]. Herein, we present 2 rare familial cases of delusional parasitosis with folie à deux and folie à trois, highlighting the clinical complexities of, diagnostic challenges of, and therapeutic approaches required for managing shared delusional infestation within family units.

## Case Report

### Ethical Considerations

This case report did not require approval from an institutional review board or ethics committee, as it is based on patient observations without experimental intervention, in accordance with institutional and local policies. Written informed consent was obtained from the patients for publication of the case details and associated images. All data were anonymized to protect the patients’ privacy and confidentiality. No compensation was provided to the patients for participation or publication.

### Family 1

**Figure 1.**
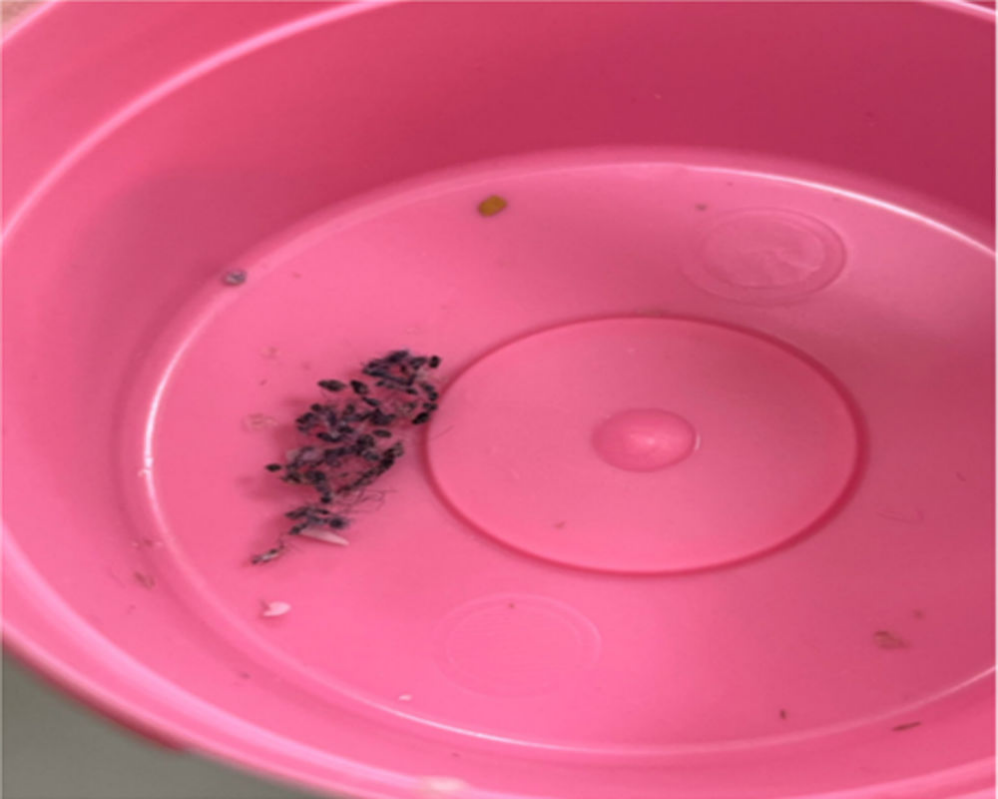
The patient presented a pink box with suspected parasitic material, which was later identified as skin debris and textile fibers.

**Figure 2.**
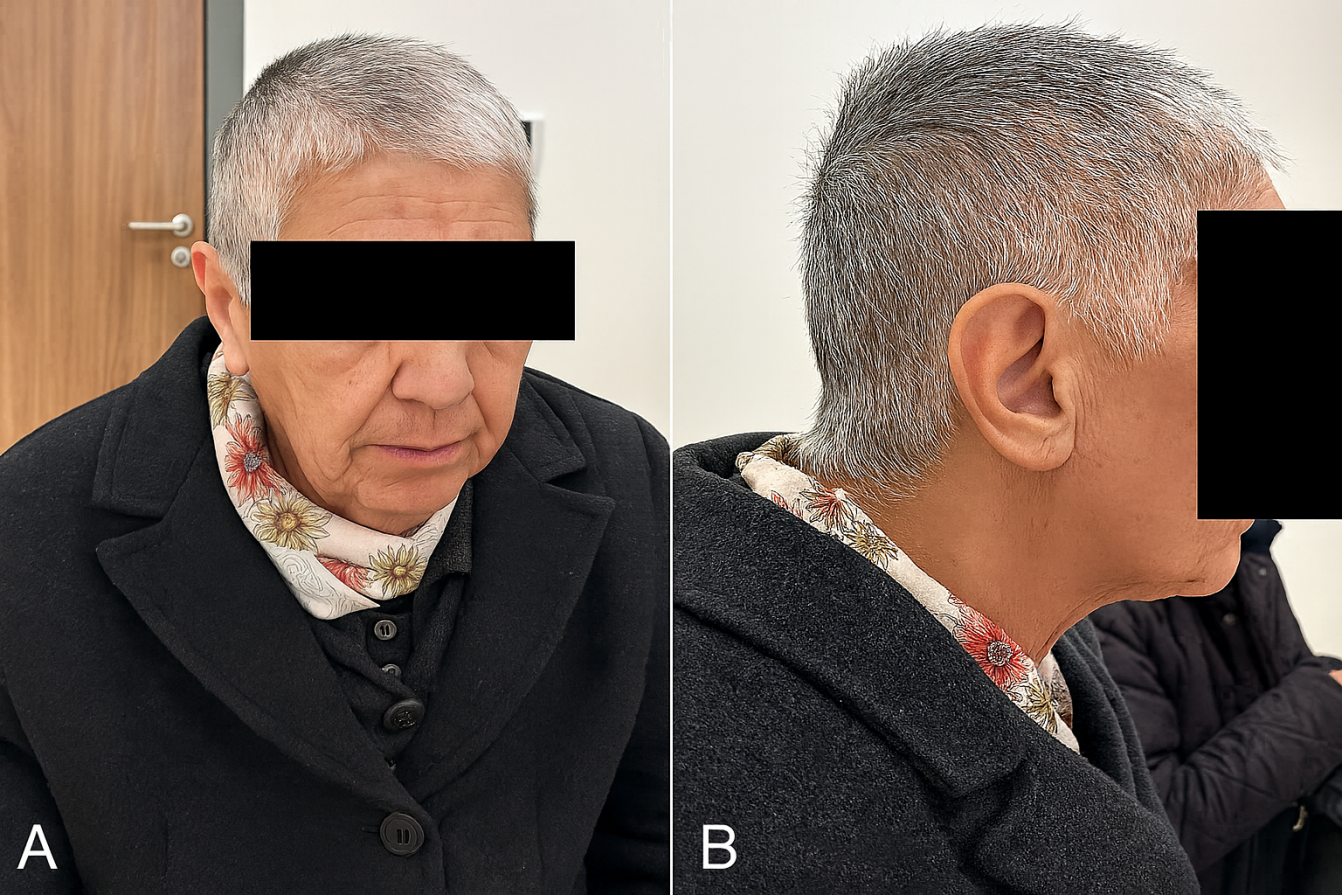
Clinical presentation of the patient from family 1. (A) Frontal view showing self-induced haircut due to persistent scalp infestation delusion. (B) Lateral view revealing no visible scalp lesions.

A 70-year-old woman presented with a 2-month history of persistent pruritus, sleep disturbances, and visual and tactile hallucinations characterized by the perception of insects crawling on her body and within her surrounding environment. The symptoms initially emerged 6 months prior to our assessment, following a scabies infestation that had been successfully treated, though it progressively worsened over time. She had sought dermatological evaluation multiple times, receiving various treatments without sustained relief. The patient reported engaging in repetitive hygiene-related behaviors, including frequent face washing, excessive body wiping, repeated hair washing, and eventually self-inflicted hair cutting. She had also developed significant anxiety and distress, believing that the infestation was spreading despite multiple self-directed treatment attempts. Upon dermatological examination, multiple linear excoriations were noted on the patient’s extremities. Additionally, she presented a box with suspected parasites, but it contained only skin debris and textile fibers (Figure 1). A clinical examination demonstrated self-induced scalp hair cutting without any visible lesions (Figure 2). A macroscopic examination of the sample provided by the patient revealed no evidence of parasitic organisms, with the observed structures consisting of skin debris and textile fibers.

Notably, the primary patient’s husband began experiencing similar symptoms 1 month after the onset of the primary patient’s condition, describing visual hallucinations of insects and the sensation of crawling on his skin. Both patients denied any prior psychiatric history, substance use, and significant medical comorbidities. However, the primary patient exhibited more severe symptoms, including functional impairment, social withdrawal, and heightened emotional distress.

Due to the persistent nature of symptoms and evidence of shared delusional beliefs, a psychiatric consultation was requested. Both the primary patient and her husband underwent the Mini-Mental State Examination (MMSE), yielding scores of 27 and 28 out of 30, respectively. These results suggest mild cognitive decline but no overt dementia. The Minnesota Multiphasic Personality Inventory (MMPI) was also administered to the patients. The primary patient’s MMPI results revealed elevated hypochondriasis and anxiety subscale scores, which are consistent with an underlying delusional disorder. For her husband, only the hysteria subscale score was elevated. Laboratory evaluation results (including complete blood counts; metabolic panels; liver and renal function tests; vitamin B12 levels; thyroid function tests; and serological tests for hepatitis, syphilis, and HIV) were all within normal limits, which ruled out organic causes. A brain magnetic resonance imaging (MRI) scan was also performed for the primary patient, revealing no structural abnormalities.

A structured management plan was initiated, for which the primary patient was started on aripiprazole at 1 mg/day. However, due to poor adherence, which was attributed to the exacerbation of pruritus and a skin rash, the treatment was switched to trifluoperazine at 1 mg/day, with a plan for gradual titration. Environmental modifications and support were provided to the primary patient’s partner, who showed mild symptom improvement with behavioral therapy alone.

### Family 2

A 59-year-old married woman—a mother of two—presented with a 1-year history of persistent pruritus and the sensation of insects crawling on her body. She had initially sought dermatological evaluation multiple times, convinced that she had pediculosis or scabies; however, a clinical examination and laboratory investigations failed to confirm any parasitic infestation. The patient had undergone various empirical treatments, including antiparasitic shampoos and repeated courses of topical medications. Despite the lack of medical confirmation, she persistently self-administered these treatments. The onset of her symptoms was temporally associated with the concern that her son’s friend, who had been recently released from prison, might have introduced parasites into their home. Despite reassuring explanations from multiple physicians, she continued to engage in compulsive hygiene behaviors, including daily house disinfection, meticulous ironing of clothes, and frequent bathing, leading to progressive social withdrawal.

**Figure 3.**
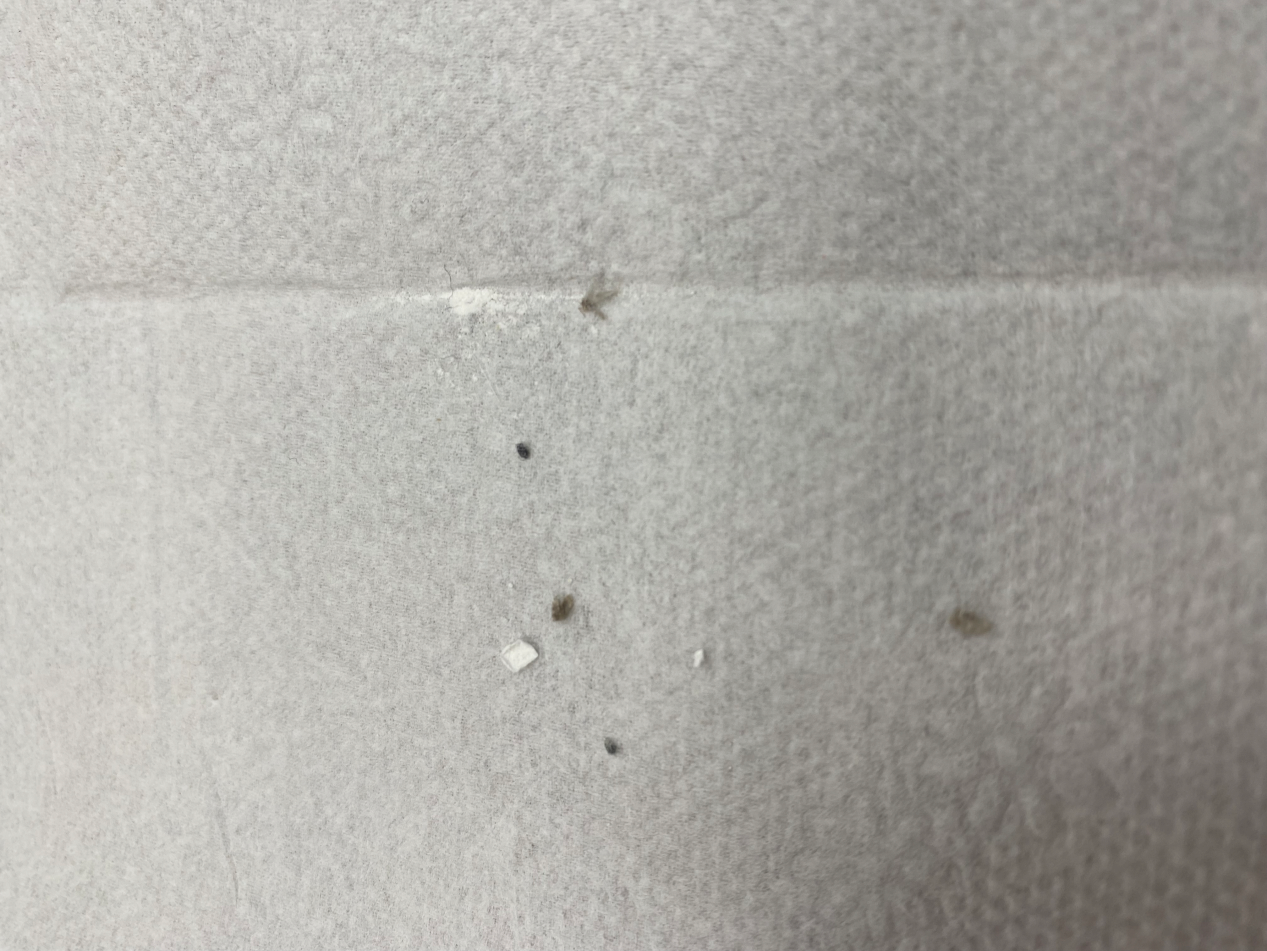
The patient provided a white napkin containing alleged parasites, which, on examination, revealed a psychodid fly.

**Figure 4.**
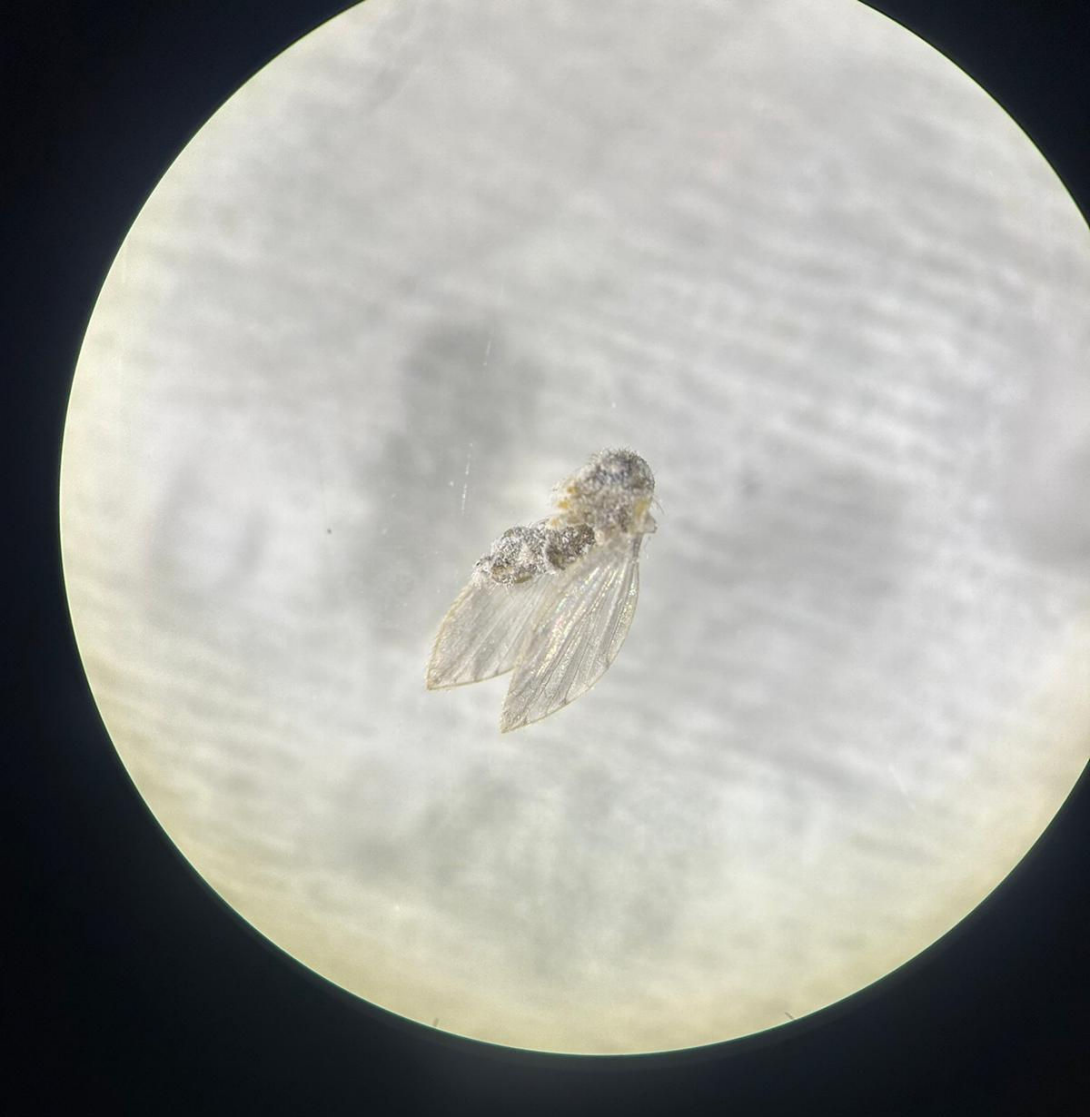
Microscopic view of the sample from Figure 3, showing a psychodid fly—a nonparasitic insect species.

Approximately 1 week after staying at the patient’s home, her sister developed similar symptoms, reporting crawling sensations, frequent washing, and self-inflicted hair cutting. Shortly thereafter, the patient’s niece, who had briefly visited the sister’s home, also developed identical symptoms, suggesting a progressive shared delusional component within the family unit. Additionally, the patient provided a sample on a white napkin, which she insisted contained evidence of the parasites (Figure 3). A microscopic evaluation identified the presence of a fly within the sample (Figure 4).

None of the affected individuals had a history of psychiatric disorders, substance abuse, or alcohol consumption. Due to the worsening clinical picture and evidence of a transmitted delusional belief, a psychiatric evaluation was conducted. The Positive and Negative Syndrome Scale (PANSS) was administered, with the index patient scoring 69 (positive symptoms subscale score: 16; negative symptoms subscale score: 12; general psychopathology subscale score: 41), indicating significant psychotic symptoms. To exclude organic causes, a comprehensive laboratory workup (including complete blood counts; serum electrolytes; liver and renal function tests; fasting glucose; thyroid function tests; vitamin B12 levels; and serological tests for hepatitis, syphilis, and HIV) was performed, and all results were within normal limits. A brain MRI scan was also conducted for the primary patient, revealing no structural abnormalities.

She was referred to the psychiatry department for a consultation, and trifluoperazine was initiated at 5 mg/day, with a planned dose escalation. Over the course of hospitalization, her pruritic symptoms gradually diminished; however, she continued to express concerns about spreading the infestation to others, reflecting partial insight impairment. Consequently, her trifluoperazine dose was increased to 15 mg/day, and structured psychotherapy was introduced, focusing on cognitive restructuring and anxiety management techniques.

Psychoeducation sessions were also conducted for the patient’s sister and niece to enhance their understanding of the disorder and minimize the reinforcement of shared delusional beliefs. They were advised on strategies for supporting the patient’s recovery while avoiding behaviors that might reinforce the delusion.

By the fourth week of treatment, the patient exhibited a significant reduction in symptom severity, with her PANSS score improving to 50 (positive symptoms subscale score: 11; negative symptoms subscale score: 12; general psychopathology subscale score: 27). After marked improvements in functionality and symptom control, she was discharged with outpatient psychiatric follow-up appointments, continued pharmacotherapy, and ongoing family psychoeducation for preventing symptom recurrence in the shared delusional network.

## Discussion

Delusional parasitosis is classified into primary, secondary, and organic forms. Primary delusional parasitosis occurs as an isolated delusional disorder without any underlying psychiatric or medical condition [7]. Secondary delusional parasitosis is associated with psychiatric disorders, such as schizophrenia, major depressive disorder, dementia, anxiety disorders, and phobias [6,8]. The organic form of delusional parasitosis arises due to medical conditions, including hypothyroidism, anemia, diabetes mellitus, vitamin B12 deficiency, hepatitis, syphilis, and HIV infection [2]. Additionally, substance abuse, particularly cocaine use, has been implicated in triggering delusional parasitosis symptoms [5]. In our cases, neurocognitive evaluations, including the MMSE and MMPI, showed no significant cognitive deficits, suggesting a primary psychiatric etiology. Further, the laboratory outcomes were within normal limits and thus excluded organic reasons. As such, our cases were accepted as primary delusional parasitosis.

A key clinical hallmark of delusional parasitosis is the matchbox sign, that is, patients presenting dermatologists with small particles, such as dust, skin debris, or fibers, as “evidence” of their infestation [9]. In our cases, patients presented us with similar materials, including dust, fibers, and skin debris. In the second case, the patient presented a fly, which was identified as belonging to the family Psychodidae and subfamily Psychodinae (flies that do not harm humans), in addition to these materials. Patients with delusional parasitosis frequently experience tactile hallucinations, including sensations of crawling, stinging, or biting, which reinforce their conviction of infestation [10]. Consequently, they often engage in excessive hygiene practices, such as repeated washing, application of caustic substances, or compulsive skin scratching, which may lead to secondary skin damage, including excoriations, ulcerations, and irritant contact dermatitis [11]. The psychological burden associated with delusional parasitosis frequently results in social withdrawal; depression; and, in some cases, self-harm (as observed in our cases) [7].

Shared psychotic disorder (folie à deux) is a rare and complex psychiatric condition in which 2 or more individuals develop the same delusional beliefs [12]. Typically, a dominant individual (the primary case) has an underlying psychotic disorder, while the secondary individual adopts the same delusion through close emotional association and suggestibility. The primary case often presents with a chronic psychotic illness, such as a delusional disorder, whereas the secondary individual typically exhibits a more passive personality, lower self-esteem, and heightened susceptibility to suggestion [13].

Shared psychotic disorder is most commonly observed within nuclear families, with delusional beliefs typically being transmitted between spouses, between siblings, or between parents and children. This indicates that both genetic predisposition and psychosocial factors play a role in the disorder’s etiology [13]. In our first case, the disorder manifested between a married couple; the wife, as the dominant individual, exhibited primary psychotic symptoms, and the husband later developed hallucinations and delusions that were similar to those of the wife. The second case involved a progressive transmission of delusional beliefs to the patient’s sister and niece, which is consistent with folie à trois, wherein 3 individuals share the same fixed delusion.

Psychosocial stressors may act as triggers or maintaining factors of delusional parasitosis [6]. In our first case, the initial scabies infestation acted as a triggering factor for the development of delusional beliefs. In contrast, the delusion in the second case was precipitated by the patient’s concern that her son’s friend, who had been a guest in their home, might have introduced parasites into the household.

The management of shared delusional parasitosis is complex, requiring both pharmacological intervention and psychotherapeutic intervention [9]. Due to the lack of large-scale randomized controlled trials that focus on delusional parasitosis, treatment strategies rely primarily on case reports and small-scale studies [11]. Antipsychotic treatment remains the cornerstone; in particular, second-generation agents, such as risperidone and olanzapine, have demonstrated efficacy in reducing symptom severity [2]. However, patient compliance remains a significant obstacle [9].

In our first case, aripiprazole—an atypical antipsychotic drug—was initiated but was later switched to trifluoperazine—a first-generation antipsychotic—because of a skin rash. Adverse reactions, such as extrapyramidal symptoms, required adjunctive biperiden therapy, which led to symptom improvement. This finding is consistent with reports describing the necessity of extrapyramidal symptom monitoring in patients with delusional parasitosis who are on first-generation antipsychotics [1]. In the second case, the patient was treated with trifluoperazine and did not exhibit extrapyramidal symptoms.

An important consideration in shared delusional parasitosis cases is whether both the inducer and the recipient require treatment. Although the primary patient typically requires pharmacological intervention, the recipient may improve with separation and psychoeducation alone [12]. In our cases, behavioral therapy and psychoeducation were sufficient for secondary patients, who exhibited spontaneous symptom remission once the inducer underwent structured treatment. Psychoeducation also played a critical role in preventing the reinforcement of delusional beliefs among family members—a strategy emphasized in previous reports [2].

## Conclusion

Shared delusional parasitosis (folie à deux and folie à trois) is a rare but clinically significant disorder that poses diagnostic challenges, particularly in cases involving family members or individuals in close relationships. Delusional parasitosis is often misdiagnosed as true parasitosis, leading to repeated antiparasitic treatments and delays in psychiatric intervention. Since dermatologists are often the first point of contact, it is crucial that they recognize delusional parasitosis early and facilitate psychiatric referrals. In our cases, patients underwent multiple dermatological consultations before receiving a psychiatric diagnosis, illustrating the need for greater awareness among dermatologists. Establishing a therapeutic alliance with patients is essential, as direct confrontation may lead to treatment refusal and the worsening of symptoms. As such, dermatologists should adopt a supportive approach and serve as a bridge between patients and psychiatric care to facilitate appropriate intervention. The management of delusional parasitosis requires close collaboration between dermatology and psychiatry professionals.

## Abbreviations

MMPI: Minnesota Multiphasic Personality Inventory
MMSE: Mini-Mental State Examination
MRI: magnetic resonance imaging
PANSS: Positive and Negative Syndrome Scale

## References

[1] Bewley AP, Lepping P, Freudenmann RW, Taylor R (2010). Delusional parasitosis: time to call it delusional infestation. Br J Dermatol.

[2] Reich A, Kwiatkowska D, Pacan P (2019). Delusions of parasitosis: an update. Dermatol Ther (Heidelb).

[3] Mumcuoglu KY, Leibovici V, Reuveni I, Bonne O (2018). Delusional parasitosis: diagnosis and treatment. Isr Med Assoc J.

[4] Kim C, Kim J, Lee M, Kang M (2003). Delusional parasitosis as 'folie a deux'. J Korean Med Sci.

[5] Lepping P, Freudenmann RW (2008). Delusional parasitosis: a new pathway for diagnosis and treatment. Clin Exp Dermatol.

[6] Szepietowski JC, Salomon J, Hrehorów E, Pacan P, Zalewska A, Sysa-Jedrzejowska A (2007). Delusional parasitosis in dermatological practice. J Eur Acad Dermatol Venereol.

[7] Al-Imam AML (2016). A systematic literature review on delusional parasitosis. Journal of Dermatology & Dermatologic Surgery.

[8] Jerrom R, Mortimer H, Martin K, Siddiquee R, Bagchi D, Goulding JMR (2020). A case series of shared delusional infestation: folie à deux revisited. Clin Exp Dermatol.

[9] Campbell EH, Elston DM, Hawthorne JD, Beckert DR (2019). Diagnosis and management of delusional parasitosis. J Am Acad Dermatol.

[10] Ahmad K, Ramsay B (2009). Delusional parasitosis: lessons learnt. Acta Derm Venereol.

[11] Gajbhiye A, Ali T, Aziz S (2023). Delusional parasitosis: a case series. Ind Psychiatry J.

[12] Giam A, Tung YL, Tibrewal P, Dhillon R, Bastiampillai T (2017). Folie à deux and delusional parasitosis. Asian J Psychiatr.

[13] Galindo C, Galindo L (2025). Folie à deux in the family environment: a case of shared delusion between a mother and her son. Cureus.

